# Auxetic Behaviour of Rigid Connected Squares

**DOI:** 10.3390/ma16155306

**Published:** 2023-07-28

**Authors:** Julian Plewa, Małgorzata Płońska, Grzegorz Junak

**Affiliations:** 1 Faculty of Science and Technology, Institute of Materials Engineering, University of Silesia in Katowice, 1a, 75 Pułku Piechoty Str., 41-500 Chorzów, Poland; malgorzata.plonska@us.edu.pl; 2 Faculty of Materials Engineering, Silesian University of Technology, 44-100 Gliwice, Poland

**Keywords:** auxetics, rotating rigid units, expansion, compression

## Abstract

The paper presents an analysis of rotating rigid unit (RRU) auxetic structures, the special property of which is negative Poisson’s ratio. The crucial features of such modified structures are the well-functioning linkages of the square units at their pivot points. This ensures the stable functioning of such structures in tension or compression. The presented geometrical analysis of these auxetic structures may facilitate their adequate construction and allow one to determine the expected values of their expansion as well as the desired porosity. The results are confirmed based on the behaviour of physical models produced by the assembly of square units. The change in the dimensions of the physical models when moving from a closed to an open position is consistent with the predictions of the geometric models. By modifying the well-known ‘rotating squares’ model, physical structures with auxetic properties are obtained that can be utilised in industrial conditions, where a simultaneous change of linear dimensions is needed—either in compression or in tension. The assembly method may prove efficient in building such structures, given the abilities of assembly robots to regularly arrange the unit cells in lines and rows and to connect them with rings at the designated positions (evenly spaced perforations). The presented auxetic structures might find their potential application in, e.g., expansion joints or structures in which the porosity is mechanically changed, such as mesoscale structures. The tested structures subjected to high compressive forces buckle when the yield strength of the rigid unit material is exceeded.

## 1. Introduction

Mechanical metamaterials are engineering materials that exhibit unusual mechanical properties, such as negative Poisson’s ratio, negative compressibility, etc., when subjected to mechanical stress.

Recently, this hitherto less significant group of metamaterials has been attracting considerable interest, particularly due to clearly defined engineering needs associated with it. These include technical solutions where the weight of a given structure needs to be reduced and where special mechanical properties need to be obtained, such as specific elastic properties, increased impact resistance, or controlled equidirectional change in the structure’s size. 

If all these properties are present in one material, an auxetic behaviour is obtained. This is particularly highlighted in numerous computational studies which enthusiastically demonstrate it by means of simulation and computer graphics, e.g., [1,2,3,4,5,6,7].

Although the models used in the simulation studies allow one to estimate the possibility of obtaining auxetic behaviour and specific mechanical properties, they pose a challenge for engineers when it comes to their implementation. Theoretically, these models can work at any scale, but they can be implemented only within a specific range, i.e., where mechanical metamaterials are deformable elastic bodies.

To produce any actual structure, one has to work with a specific material, and Poisson’s ratio can therefore be determined by the type of unit cells and their arrangement.

For square unit cells, the way in which the units are combined is decisive for the size of the structure, but it does not affect Poisson’s ratio. Simulations, on the other hand, yield theoretical relationships that result from the compensating effect of correlated physical quantities.

It can be added that theoretically, for these materials, the physical properties obtained, such as Poisson’s ratio ν, dynamic mass density ρ_eff_, and thermal expansion coefficient (CTE), can take on hitherto unseen values [8].

The existing classic works in this area include simulation studies [9,10,11,12]. The scientific world continues to focus mainly on elegant theoretical studies that often consider broader parameter changes, while even a small change is already significant since the parameters are related to the elastic properties of materials [9,10,11,13,14]. 

Theoretical considerations regarding the boundary parameter values for metamaterial structures, e.g., the characteristic angle for unit cells equal to zero (*θ* = 0° no structure) or for the zero angle (Δ*θ* = 0° locking position) changes are also not practically useful in engineering. It should be made clear that Poisson’s ratio relates to a change in the dimensions of the structure—i.e., therefore, there must be a change in the structure’s characteristic parameter, in this case, the angle *θ*, albeit by a small finite amount. It can be said that Poisson’s ratio—according to the theory of linear elasticity—applies to motion, i.e., a dynamic situation but within a narrow range of parameter change.

State-of-the-art auxetic structures still exist mainly at the theoretical level and involve digital simulations. Many such studies are conducted nowadays, although already with a significant role of topology [1,15,16,17,18], while for the rotating square-type structures, dilation is considered the dominant deformation type [1,15]. Hence, there is a considerable body of theoretical knowledge to work with.

Poisson’s ratio is used to describe the degree of lateral strain ε relative to the axial strain and has the following formula:

(1) $$\nu =-\frac{\varepsilon_{lateral}}{\varepsilon_{axial}}$$

An engineering approach is needed that would confront the theoretical insights with the possibilities and needs of technology. As these metamaterials have enormous application potential, this requires an extremely broad approach, as well as very substantial resources. 

Engineering metamaterial solutions mostly include numerous non-auxetic architectural solutions [19], technical solutions akin to origami [20], or wave springs [21], and auxetic sandwich panels with a cellular core [22,23,24,25,26,27] as well as well-known auxetic foams by Lakes [28,29,30]. Real-world metamaterials also include, for example, auxetic shoes [31], a tennis racket [32], or an adhesive elastic kinesiology tape [33]. There is also a considerable number of imaginary proposals for auxetic applications, albeit having a physical basis. Among other things, auxetic materials are expected to have synclastic curvature while bent [34], variable permeability [35], high shear stiffness [36], increased indentation resistance [36,37], high fracture toughness and high impact resistance [38,39], and sound attenuation and absorption capacity [40]. These extraordinary properties offer many potential uses for auxetic materials in biomedicine [41,42], safety equipment, cushioning materials [43], energy harvesting devices [44], tunable filters [45], sensors, soft robotics [46], fashionable textiles [47] blast-resistant protective systems [48,49,50], as well as applications in aircraft industry [51] and sports equipment [52].

In general, it is reported in the literature that auxetic materials have enhanced properties compared with conventional materials, which is demonstrated in theoretical modelling studies.

The design of metamaterials consists of a regular arrangement of unit cells that form 2D or 3D structures. Two such structures play a significant role, namely, those made up of reentrant unit cells (i.e., concave honeycomb cells) and rotating rigid units. The resulting homogenised properties of the auxetic structures (the macroscale) are, of course, highly dependent on the type of unit cells (the micro/nanoscale).

A particularly interesting group of auxetics are structures containing rigid rotating units. Materials with an NPR (Negative Poisson’s Ratio) structure with rigid rotating units in the form of squares [53], triangles [54], lozenges [54] or stars [55,56] are among the most-studied auxetics.

Inspired by the model of rotating rigid units, auxetic behaviour has been demonstrated in modified systems: either by introducing connections of single square units [57,58,59,60] or by manufacturing structures in the form of auxetic plates containing diamond-shaped perforations [61,62,63,64]. In the latter group, the rigid rotating model was further extended by the rotation of semi-rigid units.

To date, many papers have been published on the manufacturing of auxetic materials. A variety of conventional [64,65,66,67,68] and incremental manufacturing processes are employed [68,69,70,71,72,73], with the present work utilising the assembly method.

The crucial point of this work has been the need to confirm the auxetic properties of such structures, to test their expansion possibilities, and, in particular, to confirm the theoretical considerations with physical models.

As it is widely known, in most metamaterials, the maximum elastic expansion is very small, meaning that macroscopically the dimensions can only change to a limited extent. Through the modification of such structures, much greater expansion possibilities have been obtained. The production of physical models of auxetic structures can be effectively realised by assembling them from single-unit cells.

The observed progress in research and development of mechanical metamaterials is an encouragement to explore such structures in depth, build their models, and confirm their extraordinary properties. 

It should be emphasised that the auxetic structures presented herein do not utilise the properties of elastic materials but only the rigidity of the square units. In this respect, such structures are a definite novelty in this field.

The research undertaken herein has focused on the precise study of the function and feasibility of the known auxetic structures, starting with a very interesting invention by R.D. Resch from 1985 (U.S. Patent 3.202.894) popularised by Grima and Evans under the name ‘rigid rotating squares’. The structure of such rotating squares is presented below from a new perspective, with the goal of finding new potential applications.

Two-dimensional structures of interconnected rigid rotating squares are thus the focal point of the present work.

## 2. Analysis of the Rotating Squares Structure

In the original proposal by R. D. Resch and in the work of Grima and Evans [53], cells in the form of squares are connected at their vertices, which in the implementation requires these vertices to be thickened. When mechanical forces are applied to structures made of such unit cells, one can identify particularly sensitive material areas. These areas (cell connections) become particularly vulnerable to high stress and inevitably fail after several mechanical cycles.

Most auxetic structures have cell joints and pores. These joints need to be stronger and more flexible than the rest of the unit cell. Due to the applied stresses, cell joints become particularly susceptible to cyclic fatigue loading. 

In a modified proposal [57,58], square unit cells are connected at the pivot points located on their surface. This solution ensures the continuous and failsafe functioning of such physical structures. Thus, it may have a wide range of potential applications.

**Figure 1:**
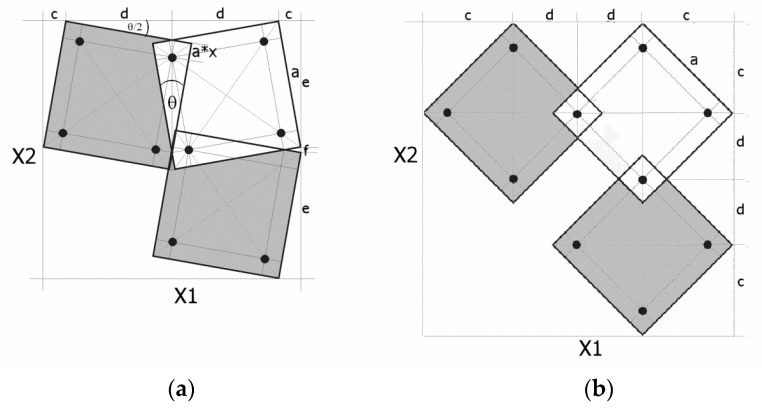
A structure of connected squares in the *closed* position (**a**) and in the *open* position (**b**), with the characteristic sizes indicated.

The position of the pivot points is defined by the geometric parameter x, whereby the distance between the edge of the square and the pivot point located on its diagonal is equal to a × x, where a is the length of the side of the square (Figure 1).

The parameter x can vary between 0 < x < 0.25. For a value of x = 0, a theoretical structure is obtained in which the squares contact each other with their vertices, while for x = 0.25, the squares overlap so far that they completely block each other, and no rotational movement at the pivots is possible. The parameter x has become the key design parameter allowing for the adjustment of the desired properties of the produced structures.

By assembling square unit cells connected at the pivot points, one can build auxetic structures of any size. Their characteristic features are the geometrical relationships that allow for the determination of all technically relevant parameters, namely, the *θ* angle (for a *closed* structure), the horizontal X1 and vertical X2 sizes, as well as the porosity. This allows for the extent of the structure’s expansion and compression to be calculated, as well as for the change in the size and surface area of the pores created by stretching the structure. It is also possible to determine the changes in the position of the pivot points and the contact length of the squares in full compression (*closed* position). By designating the maximal stretching of the structure as the *open* position and its maximal compression as the closed position, one can obtain all their characteristic values.

In the *closed* position, the angle *θ* formed between the edges of the squares is expressed by the following Formula (2) [57].

(2) $$\tan\frac{\theta }{2}=\frac{1-\sqrt{1-4\times x}}{1+\sqrt{1-4\times x}}$$

The effect of the parameter x on the value of the angle *θ* in the closed position can be obtained from Formula (2). This relationship is particularly useful in the construction of an auxetic structure.

Using geometric analysis, on the other hand, it is possible to derive the exact expressions for the length X1 and height X2 of the structure, given the lengths of the individual component sections. Table 1 summarises the relationships that allow for the calculation of the structure’s dimensions [57,58]. Based on the identified relationships, all the characteristic parameters of such a structure can be determined.

By summing up all the section lengths, the dimensions of the structure are obtained, first for the *closed* position, i.e., for the angle *θ*, given by the geometric parameter x, and then for the *open* position (the angle between the edges reaching 90°). This makes it possible to determine the change in the dimensions of the structure. The described transition represents the movement of the structure and corresponds to the action of a mechanical stimulus on it, leading to an increase in the size of the structure.

For a modified structure, this transition requires little energy.

The expansion can be expressed in terms of the relative change in dimensions for the extreme positions:

(3) $$\frac{\Delta X1}{X1}=\frac{X1\left(open\right)-X1(close)}{X1(close)}$$

(4) $$\frac{\Delta X2}{X2}=\frac{X2\left(open\right)-X2(close)}{X2(close)}\;$$

The ratio of the relative change in dimensions (3) and (4) with a negative sign gives the value of Poisson’s ratio, which can be calculated from the sum of the values given by the formulae in Table 1 or from direct measurements of the sections’ length.

**Figure 2:**
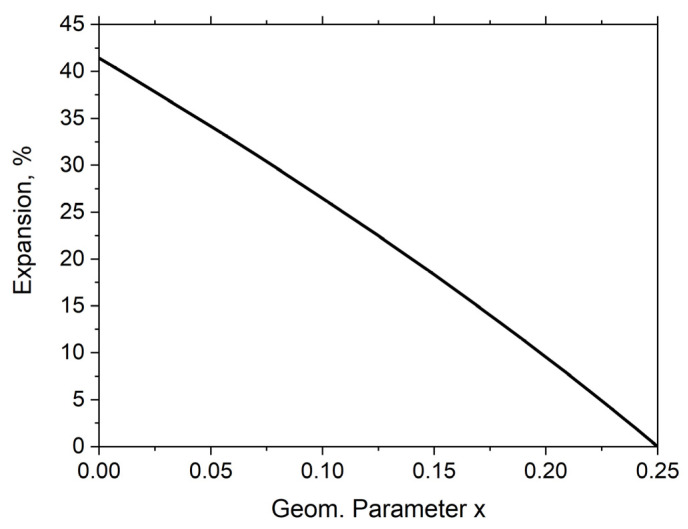
Expansion $\frac{\Delta X}{X}$, % as a function of the geometric parameter x.

The relative change in the linear dimensions of the structure in tension is approximately 41.4% for x = 0 and drops to 0 for x = 0.25 (Figure 2).

The following equation was fitted to the expansion curve:

y = −1.1247 × 10^2^x^2^ − 1.3688 × 10^2^x + 4.1352 × 10^1^, R^2^ = 9.9998 × 10^−1^ (5)

The above equation can be used to calculate the expansion for a given value of the parameter x, which is decisive for determining the maximum change in size. Unit cell displacement is a function of the parameter x.

The presented relationship indicates that structures made of square unit cells can exhibit large changes in size without any plastic deformation of the rigid square material. 

It should be added that the structures under consideration, made up of square units, satisfy the following condition:

(6) $$\frac{\Delta X1}{X1}=\frac{\Delta X2}{X2}\;$$

This means that for structures made up of rotating rigid square unit cells, there exists an auxetic condition expressed by Poisson’s ratio remaining constant at −1 and independent of either the change in the dimensions or the number of elements.

(7) $$\nu_{12}=-\frac{\frac{\Delta X1}{X1}}{\frac{\Delta X2}{X2}}=-1$$

A theoretical analysis based on the calculation of X1 and X2 in the closed and open positions shows that the relative changes in the dimensions from closed to open and vice versa are always the same. This is independent of the number of unit cells of the structure. This results in a Poisson’s ratio value of −1. The same result is obtained by calculating the relative change in the dimensions from length measurements X1 and X2 expressed in mm.

It is noteworthy that Poisson’s ratio determined in this way does not depend on the elastic properties of the material (e.g., the material moduli), although it is required for the proper functioning of the structure with the square unit cells made of rigid material. Moreover, the mechanical and physical properties of such structures are not related to each other. Although the elastic modulus and material density of square unit cells play an important role, they are not directly related to Poisson’s ratio.

Formula (7) shows that Poisson’s ratio of such structures is not influenced by either the x parameter, the angle θ, or the number of unit cells.

The lack of influence of deformation magnitude on Poisson’s ratio is an unusual feature of such structures, although it is not possible to change its value for square cells.

It can also be added that for a given value of the parameter x, one gets an expansion from zero to the value given by Equation (5), within which Poisson’s ratio remains constant, i.e., independent of the applied deformation. 

Auxetic structures based on rigid squares can theoretically reach an expansion of up to approx. 41.2%, but in practice, this value is usually lower since the value of the geometric parameter is x > 0.

**Figure 3:**
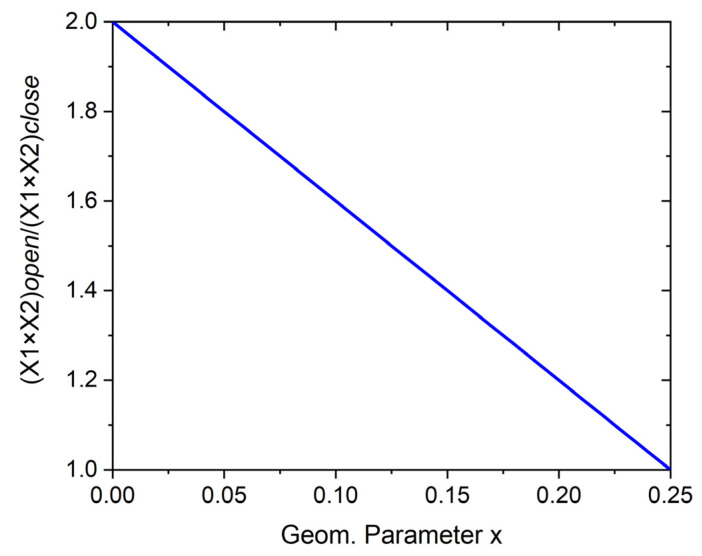
Change in the total area occupied by the structure between the *open* and *closed* positions as a function of the parameter x.

The total area occupied by the structure, expressed by X1 × X2, varies according to the value of the parameter x. Given the ratio (X1 × X2)*open*/(X1 × X2)*closed*, a straight line with a slope of −4 is obtained (Figure 3).

The total area of the structure is the area of the square units plus the area of the pores in the *open* position. The number of pores in the structure remains constant until the structure reaches the *closed* position in which all the pores disappear.

**Figure 4:**
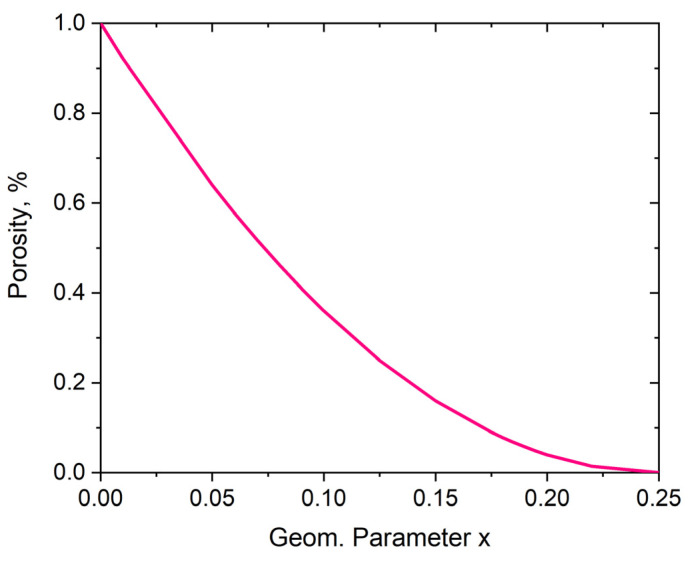
Variation of the structure’s porosity as a function of the parameter x. (y = (4x − 1)^2^).

In addition to the expansion of the structure, there is an increase in the size of the pores, i.e., the structure’s porosity, which occurs when moving from the closed to the open position. Figure 4 shows the change in the structure’s porosity as a function of the parameter x in relation to the porosity for x = 0.

The above relationship allows for the porosity variable of the structure to be modelled by manipulating the geometric parameter x. It is, therefore, possible to set a desired porosity value for the structure. By adjusting the position of the pivot points, the possible level of porosity is determined. One can, therefore, speak of modelling the porosity of such structures and, thus, of the topology of a system made up of square units.

**Figure 5:**
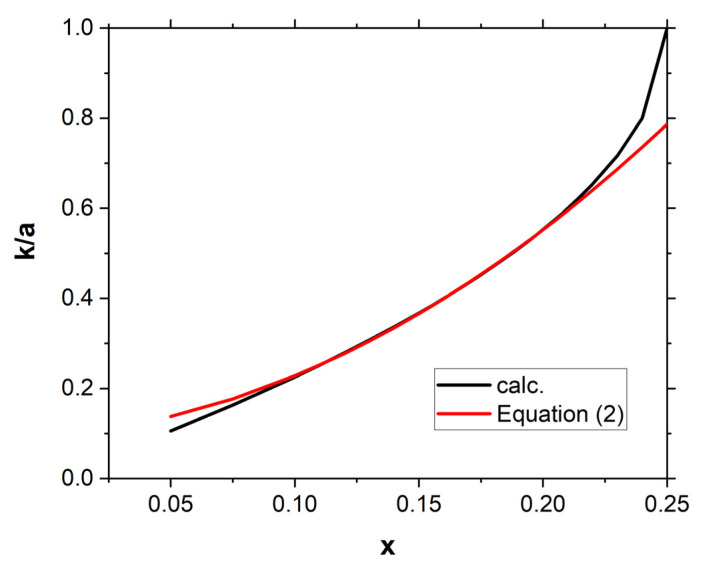
Change in the ratio k/a as a function of the parameter x.

In the *closed* position, the square cells are in contact with each other on a section of length k, where k > 2a × x. These parts of the edges of the squares, together with the pivot points, participate in the transfer of the stresses. As the parameter x increases, the contact length k increases as well. This problem may be relevant when large compressive forces act on such a structure (Figure 5).

For the curve shown in Figure 5, the following equation has been derived:

(8) $$\frac{k}{a}=9.592x^{2}+0.366x+0.095$$

For a given value of the side length *a* of a square unit cell, there is a precisely defined value of the contact length of the squares in the *closed* position. Each square’s edges are in contact with its four neighbours. The outside squares, however, only have two contacts each. These edge states only appear in the *closed* position. When pressure is applied, it is through these outer edges of the squares that the stress is transmitted. This is particularly apparent when attempting to compress the structure. 

It follows from Equation (8) that, in the closed position, the contact length of the square edges increases as the parameter x increases. This can be easily verified in a real structure in its closed position with the square unit cells in contact with each other.

The presented theoretical analysis of the auxetic behaviour of the structure is most crucially based on the geometry of square unit cells, but it is also independent of their periodicity. This means that it is universal for a structure consisting of any number of square cells connected at the pivot points.

## 3. A Study of Planar Constructions Made of Auxetic Structures

In order to better see the possibilities offered by the modified structures of the rotating squares, examples are presented below, comparing the theoretical relationships with the values obtained in the course of the experiment.

**Figure 6:**
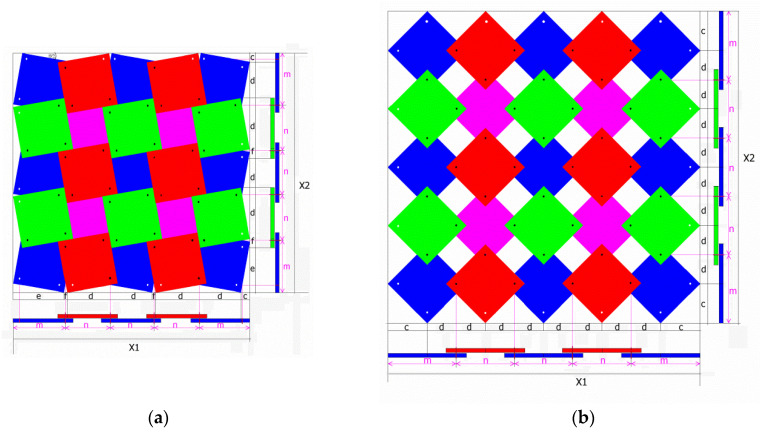
A 5 × 5 structure in the (**a**) *closed* and (**b**) *open* positions [57].

A structure made up of 25 unit cells is taken as the base (Figure 6). In tension, the unit cells move away from each other, and the structure expands in all directions.

The rigid square units are not deformed in relation to the connections between them (the hinges). The presented figures show that in the *closed* position, the edge of the first upper square forms an angle *θ*/2 with the horizontal line. They also include the characteristic values: the lengths of the component sections and the distances between the pivot points. By determining the analytical relationships of these values, a method for calculating the theoretical length X1 and height X2 of the structure has been devised.

Based on the relationships given in Table 1, one can derive the equations for the size of the structure in the *closed* position and in the *open* position (Table 2).

For the presented structure, the value of the geometric parameter is x = 0.1, which corresponds to the angle *θ*/2 = 7.23°. It should be noted that in order to determine the X1 and X2 sizes of the structure at a given moment between the extreme positions (*closed* and *open*), it is necessary to know the current value of the opening angle β, in this case, falling between β = 14.46° and β = 90°. The closed position satisfies the condition *θ* = β.

**Figure 7:**
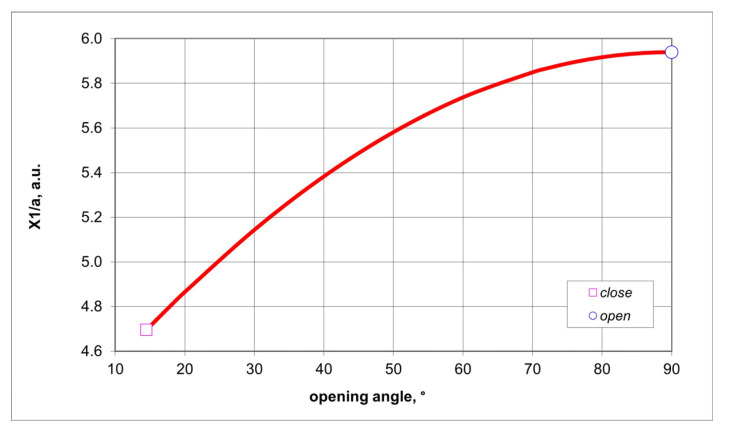
Change in the linear dimensions of a 5 × 5 auxetic structure, for x = 0.1.

The presented relationship (Figure 7), of the change in the linear dimensions (related to the length of the square side) indicates that when the structure moves from the *closed* to the *open* position, its dimensions vary along a curve. The most dynamic changes occur when a closed structure is opened.

The change of the linear dimensions between the extreme positions can be calculated from the following relationship:

(9) $$\frac{\Delta X1}{X1}=\frac{X1\left(open\right)-X1(close)}{X1(close)}$$

It should be added that for a symmetrical structure, X1 = X2 occurs. 

For the structure under consideration, one gets the following value:

(10) $$\frac{\Delta X1}{X1}=\frac{5.9396-4.69574}{4.6957}=0.2755$$

**Figure 8:**
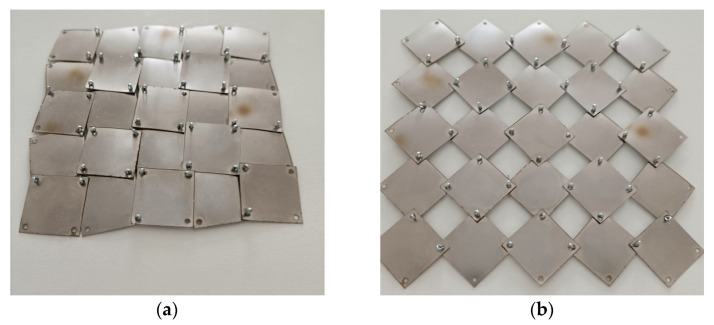
Actual 5 × 5 auxetic structures cut from steel sheet in the (**a**) *closed* and (**b**) *open* positions.

These results indicate that the transition between the closed and open positions leads to a 27.5% expansion of the structure. A physical model of the structure under consideration is presented below (Figure 8).

For the 5 × 5 structure (a = 20 mm, x = 0.1) shown in Figure 8, the following values of the relative change of dimensions have been calculated:

(11) $$\frac{\Delta X1}{X1}=\frac{120-95}{95}=0.2631$$

This symmetrical structure (5 × 5) satisfies condition (6), and its Poisson’s ratio remains constant at −1.

The theoretical value of the relative porosity for x = 0.1 is 36%. Considering that for x = 0, the pore area is 20 × 20 × 16 mm^2^, while for x = 0.1, it is 12 × 12 × 16 mm^2^—which agrees with the theoretical value.

For the *closed* position, the forces applied to the structure are transmitted through the contacting edges of the squares. The length k determined experimentally is k = 5 mm, which agrees with the value calculated from Equation (8) for x = 0.1.

**Figure 9:**
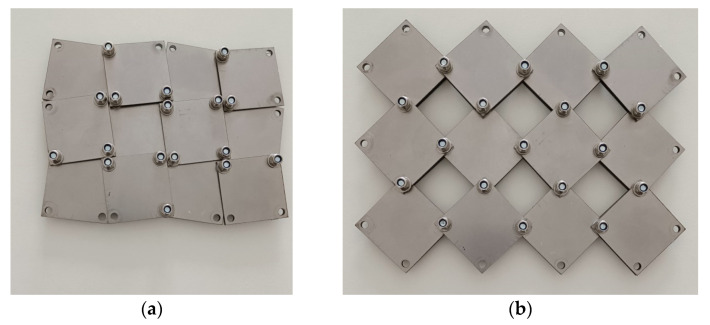
Actual 4 × 3 auxetic structures cut from steel sheet in the *closed* (**a**) and *open* (**b**) positions.

Similar observations can be made for the design of a nonsymmetrical structure (Figure 9).

Considering the asymmetrical structure (4 × 3, a = 40 mm, x = 4/40) presented in Figure 9, the following values of the relative size change have been obtained:

$$\frac{\Delta X1}{X1}=\frac{188-147}{147}=0.2789\;\frac{\Delta X2}{X2}=\frac{151-118}{118}=0.2796$$

This equation returns a Poisson’s ratio slightly below −1, which falls within the margin of error. This is due to the actual physical dimensions of the elements of the structure. Although the set value for the distance of the pivot point from the edge was x × a = 4 mm, the diameter of the pivot was also 4 mm. This created the observed difference from the theoretical value.

**Figure 10:**
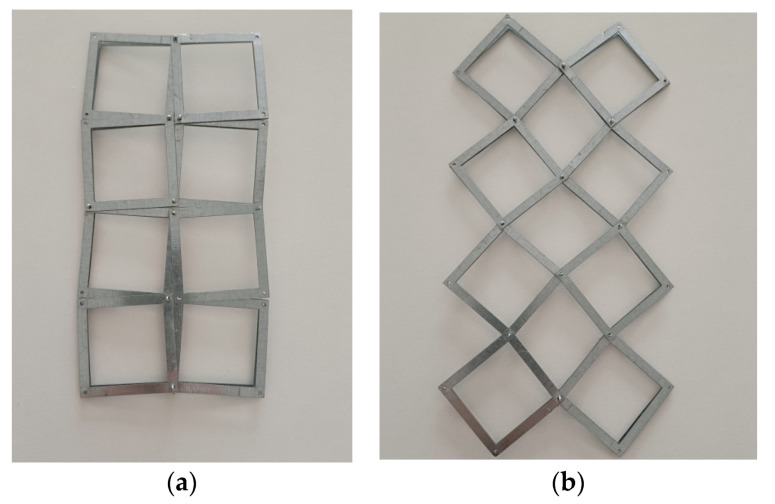
Actual 2 × 4 auxetic structures cut from an aluminium sheet in the *closed* (**a**) and *open* (**b**) positions.

The same innovative solution can be applied to a structure of connected square frames in the form of pivots on their surface (at a given distance from the edge of the square frame). These pivots located on the surface of the frames, close to their corners, allow for easy rotation almost without friction (Figure 10).

The replacement of solid squares with square frames leads to a thinner structure and allows to produce lattice structures (Figure 10). Such structures made of square frames also confirm the preliminary considerations and the derived relationships. The obtained novel structure and the assembled models can be considered a new proposal for the market of auxetic materials. The design freedom and the possibility of mass production by assembling simple components with minimal waste are the main advantages of this approach to auxetic materials.

## 4. Studies of the Deformation Mechanism of the Structure in Compression

**Figure 11:**
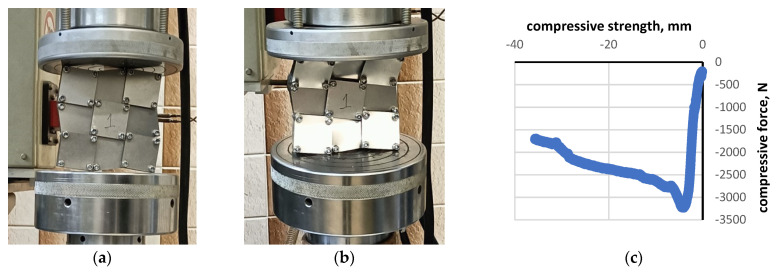
Typical mechanical compression test for a structure made of square steel units (**a**,**b**) and the stress-strain curve (**c**).

Using a universal testing machine, the 3 × 3 structure was subjected to compression tests (Figure 11). As a result of the compressive force, the structure was subjected to stress, with the transfer of stress occurring both at the contacting edges of the squares and through the pivot points. It was found that, in systems with locked cells, the mechanical stiffness at the macroscale was directly related to the number of contact points between the unit cells. The basic material properties of the unit cells play a secondary role as long as the square cells remain rigid. When the critical stress value is exceeded, the square units undergo plastic deformation, which manifests itself as buckling of the structure.

The measured displacement curves (‘controlled displacement’ in Figure 11) show an initially linear regime, with small deformations corresponding to the elastic behaviour of the locked cell structure. As the stress increases, nonlinear buckling is observed. This flat structure is not resistant to the buckling that results from the bending of the square units.

**Figure 12:**
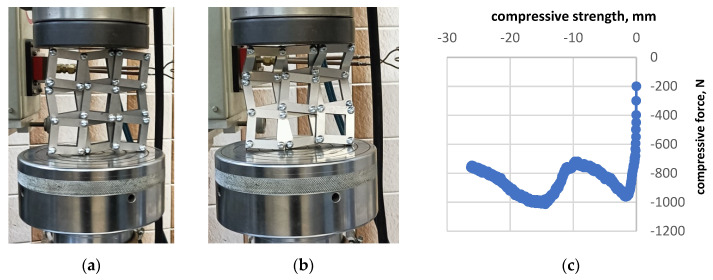
Typical mechanical compression test for a structure made of square aluminium frames (**a**,**b**) and the stress-strain curve (**c**).

Similar mechanical tests were carried out for a structure made of square frames (Figure 12).

The compression test results presented for a structure made of square frames are similar to those obtained for a structure made of full squares. There is a definite difference in the elasticity range, which is very narrow for a structure made of aluminium frames. Here, too, it is a matter of stress transfer through the contacting edges of the frames. The buckling that occurs in compression can be regarded as structural instability, which can be prevented by reducing the dimensions of the square units.

## 5. Discussion

The results of the compression tests of the structures presented above were performed for relatively high stresses to demonstrate a possible failure of such an auxetic structure. With low values of stress sufficient for the structure to move from *closed* to *open* position and vice versa, the assembled structures are not deformed in any way. In this case, one can conclude that auxetic structures formed from rigid squares connected at the pivots represent an engineering solution characterised by stability and durability—i.e., they can exhibit multiple cyclic changes in their size without breaking down. These changes are associated with a Poisson’s ratio of −1.

The presented geometrical analysis might become useful for constructors, allowing structures to be designed so that they correspond to the desired amount of maximum expansion. By defining the geometric parameter x (related to the distance of the pivot from the edge of the square unit), it is possible to determine the expected properties of an auxetic structure. In addition to the magnitude of the expansion, one can also manipulate the porosity of the structure and the size of the contact points between the square units. All these parameters derived theoretically have been reproduced in practical models using auxetic structures with rigid square units.

In this way, the presented approach can be used to reverse-calculate the structure’s micro-architecture, including lattice structures, to obtain the expected magnitude of size change in compression or tension. This applies to a stress range that does not lead to buckling of the structure. 

The results of the compression tests show the usage limits of the tested structures in compression. The typical relationships obtained from the measurements made with the testing machine show the yield strength of the tested structure, which is not lower than the yield strength for the material of the square units.

Actual physical auxetic structures built from rigid square units connected at the pivot points can be manufactured through the assembly method, which, due to its simplicity and the small number of elements, can be implemented quite easily using automated production lines. Assembling structures from individual elements (square units, pivot axes, and their fixing elements) can offer certain advantages, such as their precise positioning and adapting the material properties of the elements to specific applications.

In conclusion, it should be emphasised that the studied auxetic structures constitute real auxetic systems capable of functioning in practice and of cyclical failure-free expansion and compression. The analysis of the improved structures made of rotating squares allows for a theoretical determination of the magnitude of expansion for the given values of the length of the side of the square and the geometrical parameter x (related to the position of the pivot points). This offers a good starting point for the implementation and construction of the proposed auxetic structures. Furthermore, the number of square unit cells in the structure for the assumed dimensions X1 and X2 can also be calculated using the relationships provided in Table 1.

In summary, this paper has presented a novel design of mechanical metamaterials in which innovative topological techniques can be utilised and which can be regarded as a proposal for potential applications, for example, in expansion joints or structures with mechanically adjusted porosity, e.g., in mesoscale structures.

## Figures and Tables

**Table 1:** Expressions for the dimensions of a structure made up of squares connected at the pivot points in the *closed* position and in the *open* position [57].

*closed*	$a\times \sin\frac{\theta }{2}$	$\frac{a\times \cos\frac{\theta }{2}}{1+\tan\frac{\theta }{2}}$	$a\times \cos\frac{\theta }{2}$	$2a\frac{\tan\frac{\theta }{2}\sin\frac{\theta }{2}}{1+\tan\frac{\theta }{2}}$
*open*	$\frac{a}{\sqrt{2}}$	$\frac{a\times (1-2x)}{\sqrt{2}}$		

The theta angle applies only to structures in the *closed* position.

**Table 2:** Equations for the length and height of the structure are shown in Figure 6.

*Closed* Position	*Open* Position
X1 = e + 4d + 2f + c lub X1 = 3e + 3c	X1 = 2c + 8d
X1 = c + 4d + 2f + e	X1 = 2c + 8d

## Data Availability

Data have contained within the article.
